# Rewiring mitochondrial metabolism to counteract exhaustion of CAR-T cells

**DOI:** 10.1186/s13045-022-01255-x

**Published:** 2022-03-28

**Authors:** Yue Huang, Xiaohui Si, Mi Shao, Xinyi Teng, Gang Xiao, He Huang

**Affiliations:** 1 Bone Marrow Transplantation Center, The First Affiliated Hospital, School of Medicine, Zhejiang University, No. 79 Qingchun Road, Hangzhou, China; 2 Liangzhu Laboratory, Zhejiang University Medical Center, 1369 West Wenyi Road, Hangzhou, China; 3 Institute of Hematology, Zhejiang University, Hangzhou, China; 4 Zhejiang Province Engineering Laboratory for Stem Cell and Immunity Therapy, Hangzhou, China; 5 Institute of Immunology, Zhejiang University, Hangzhou, China

**Keywords:** CAR-T cell exhaustion, Mitochondria, Metabolism, Single-cell techniques

## Abstract

Short persistence and early exhaustion of T cells are major limits to the efficacy and broad application of immunotherapy. Exhausted T and chimeric antigen receptor (CAR)-T cells upregulate expression of genes associated with terminated T cell differentiation, aerobic glycolysis and apoptosis. Among cell exhaustion characteristics, impaired mitochondrial function and dynamics are considered hallmarks. Here, we review the mitochondrial characteristics of exhausted T cells and particularly discuss different aspects of mitochondrial metabolism and plasticity. Furthermore, we propose a novel strategy of rewiring mitochondrial metabolism to emancipate T cells from exhaustion and of targeting mitochondrial plasticity to boost CAR-T cell therapy efficacy.

## Introduction

Chimeric antigen receptor T (CAR-T) cell therapy, a paradigm-shifting arsenal in cancer treatment, has led to tremendous clinical successes for patients with hematological malignancies. The remarkable responses of relapsed/refractory patients to CAR-T cell therapy have led to broad testing of this strategy in clinical trials and increasing research within the field [1]. However, more than 50% of patients experience inferior efficacy or disease progression due to loss of antigen density, tumor microenvironment suppression and/or intrinsic CAR-T cell exhaustion [2–5]. CAR-T cell exhaustion is a vital barrier to adoptive cell therapy (ACT) and is characterized by the gradual loss of induced cytotoxicity and proliferation, sustained expression of inhibitory receptors, an increased apoptosis rate and metabolic dysfunction [6].

Mitochondria, as dynamic and interconnected organelles, play critical roles in the activation, proliferation, differentiation and effector function of T cells [7]. Impaired mitochondrial function and dynamics are key characteristics of exhausted T cells. Studies have shown that during chronic antigen stimulation, structurally defective and depolarized mitochondria accumulate in CD8^+^ T cells, and this accumulation is accompanied by a profound increase in reactive oxygen species (ROS) production, leading to defective oxidative phosphorylation (OXPHOS) and T cell exhaustion [8]. The choice of CAR signaling domains and cell culture condition adjustments affect the frequency of central memory T (T_CM_) cells, degree of mitochondrial biogenesis, and fatty acid oxidation (FAO) rate, which influence CAR-T cell proliferative capacity and survival and patient treatment efficacy [9–11]. Genetic or pharmacological modification can counteract exhaustion by changing the mitochondrial metabolism of CAR-T cells. Nevertheless, the mechanisms by which mitochondrial metabolism affects CAR-T cell survival, functionality, and differentiation remain unknown.

Herein, we review the mitochondrial characteristics of exhausted T cells and particularly discuss several aspects of mitochondrial function and dynamics in regulating T cell fate decisions and antitumor function.

## Characteristics of exhausted T cells

The T cell population comprises naïve (T_N_), memory (T_M_), effector (T_E_) and regulatory (Treg) T cells. T_N_ cells preferentially undergo oxidative phosphorylation (OXPHOS) to meet energy demands during maturation and persistence, while T_E_ cells rely mainly on glycolysis to meet biosynthetic needs. T_M_ cells undergo accelerated mitochondria-mediated OXPHOS and FAO and slowed glycolysis and preferentially exhibit mitochondrial fusion and increased mitochondrial biogenesis, supporting the rapid transition to an activated state during antigen rechallenge [12]. When activated, metabolic switching regulated by the PI3K–PKB (AKT)–mTOR pathway supports the differentiation of T_N_ cells to T_E_ cells, which rely on aerobic glycolysis to boost biosynthesis for rapid clonal expansion and cytotoxic effects [13, 14]. However, upon persistent antigen stimulation, CD8^+^ T cells enter an altered differentiation state, also known as cell exhaustion or dysfunction [15].

**Fig. 1:**
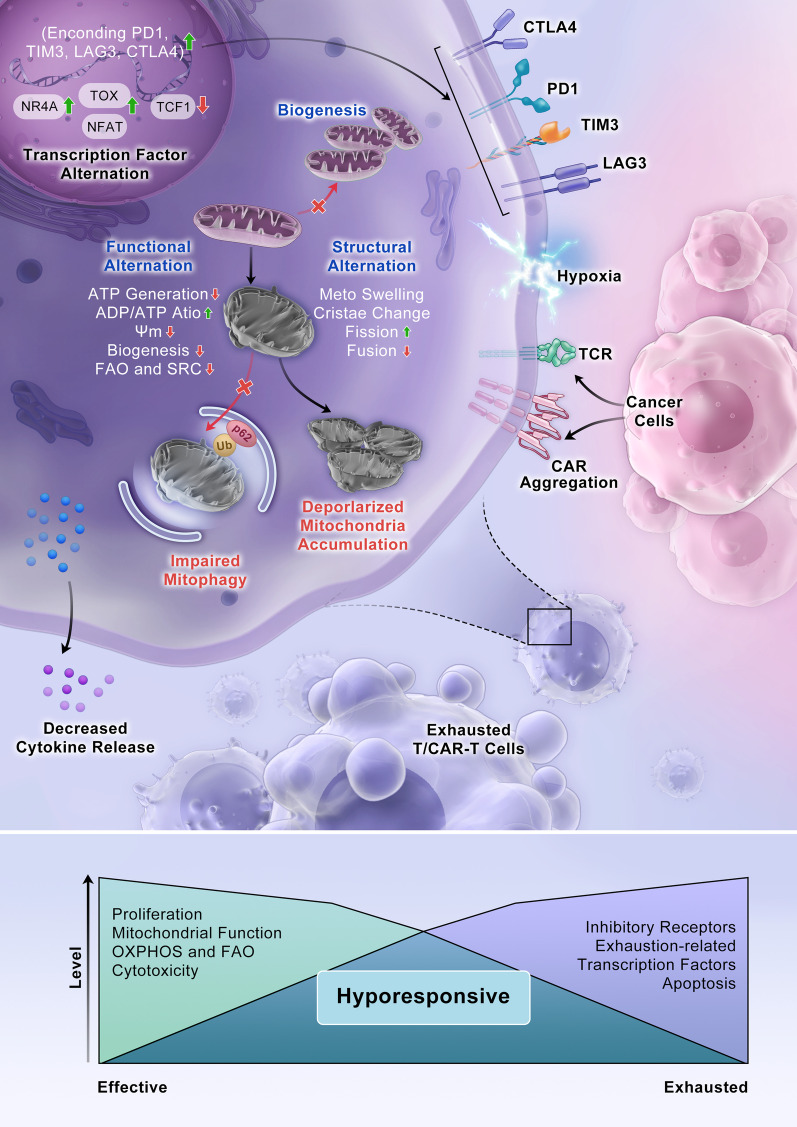
Features of T cell exhaustion and mitochondrial alterations. During persistent antigen stimulation and under hypoxia during chronic virus infection or cancer, CD8^+^ T cells enter a state of exhaustion. Tonic signaling in CAR-T cells is induced by autologous physical interactions between CAR receptors. Exhausted T cells and CAR-T cells exhibit decreased cytokine production and reduced proliferation capability; persistently high expression of multiple inhibitory receptors, such as PD-1, TIM-3, LAG-3 and CTLA4; and altered transcriptional landscapes, such as changes in changes in NR4A, TOX, TCF1 and NFAT transcription. Mitochondrial reprogramming characteristics in T cells include both functional and structural alterations. Mitochondria in exhausted cells are swollen and undergo increased fission. The mitochondrial cristae are slightly wider and more loosely organized in intermembrane regions. Functional alterations are characterized by an increased ADP/ATP ratio, decreased ATP generation and mitochondrial biogenesis, and decelerated fatty acid oxidation (FAO) and oxidative phosphorylation (OXPHOS)

A multitude of studies have revealed that the exhausted T cell population is heterogeneous, comprising cells in unique differentiation and functional states (Fig. 1). Two distinct subsets have been generally characterized: a progenitor subset (typically PD-1^hi^TIM-3^low^TCF^+^ cells) and a terminally differentiated subset (typically PD-1^hi^TIM-3^hi^TCF^−^ cells) [16, 17]. PD-1^+^TCF^+^ exhausted progenitor cells exhibit the potential to proliferate, undergo self-renewal and produce terminally differentiated exhausted cells and are critical for a proliferative burst in anti-PD-1 therapy [18]. The exhaustion of T_E_ and T_M_ cells does not follow a stepwise process; in contrast, T cell exhaustion is based on a parallel program that can be induced in T cell populations in any differentiation state. T cells in an exhausted state produce progeny with exhaustion characteristics.

In chimeric antigen receptor (CAR) T cell therapy, CARs are synthetic receptors that are designed to drive cell activation and induce antigen-specific cytotoxicity when an antigen binds to its cognate single-chain variable fragment (scFv). However, basal CAR CD3ζ phosphorylation, also known as tonic signaling, can induce early exhaustion of CAR-T cells and result in limited antitumor efficacy [19]. Exhausted CAR-T cells exhibit characteristics that are more similar to those of exhausted conventional CD8^+^ T cells than to T_E_ or T_M_ cell subsets [20]. Specifically, exhausted CAR-T cells exhibit altered effector functions, such as decreased cytokine production and reduced proliferation capability; persistently high expression of various inhibitory receptors, including PD-1, TIM-3, LAG-3 and CTLA-4; altered transcriptional landscapes, such as changed NR4A [21], TOX [22, 23], TCF1 [24] and NFAT transcription levels [25]; dramatically increased epigenetic remodeling; and metabolic reprogramming. Tonic signaling is induced by autologous physical interactions between CARs, which are antigen-independent and lead to receptor self-association and thus to CAR aggregation on the cell surface [2]. Upon tonic activation, most scFv-based CARs, such as the GD2-CAR and CD22-CAR, induce varying degrees of early T cell exhaustion. Therefore, CAR-T cells are more likely than conventional T cells to enter an exhaustion state.

## Clinical manifestations of CAR-T cell exhaustion

The clinical features of CAR-T cell exhaustion have been thoroughly investigated. Reports have indicated that the costimulatory domain and initial phenotype of CAR-T cell products influence CAR-T cell persistence and cytotoxicity. According to a single-center clinical trial, the median in vivo persistence of CD28/CD3-z CAR-T cells was 30 days, and 68 days after infusion, CAR-T cells were terminally exhausted in all patients [26]. In contrast, tisagenlecleucel, a 4-1BB/CD3-z CAR-T cell product, persisted in peripheral blood with a median time of 168 days (a range of 20–617 days) [27]. Our center reported that 4-1BB/CD3-z CAR-T cells remained viable for as long as 7 months in a Chinese patient population, confirming that CAR-T cells expressing a 4-1BB costimulatory domain persist longer than CAR-T cells not expressing this domain [28].

Initial cell phenotypes may influence the proliferation capacity and killing function of CAR-T cells, and these influences may inform treatment prognosis. Disfunction-related cell surface markers such as PD-1, LAG-3, TiM-3 and NF-α are major prediction factors. Finney et al. [29] categorized 43 young patients in a phase I clinical trial into a dysfunctional response group and a functional response group. When comparing apheresis-derived T cell material of the two groups, the research group observed a significantly higher percentage of CD8^+^ T cells expressing PD-1 and LAG-3 in the dysfunctional response group than in the functional response group, suggesting that treatment failure and short-term recurrence were closely related to initial cell phenotype before infusion. Moreover, the portion of memory-like CAR-T cells among the CAR-T products was closely related to the clinical response to CAR-T cell therapy [30]. A lower proportion of memory stem cell-like CAR-T (CAR-T_SCM_) cells may suggest CAR-T cell proliferation dysfunction and predict early recurrence of the disease [31].

## Mitochondrial reprograming in T cells

### Mitochondrial dynamics implicate metabolic activity and indicate an exhausted T cell state

Persistent antigen stimulation, hypoxia in the tumor microenvironment or inhibitory signaling can cause T cell or CAR-T cell mitochondrial alterations, including abnormal localization, morphology, structure and/or membrane potential (ΔΨm) [32, 33]. These alterations are closely related to functional damage, such as decreased ATP generation, accumulation of mitochondrial reactive oxygen species (mtROS), inhibited mitochondrial biogenesis, disrupted mitophagy and diminished mitochondria-mediated OXPHOS and FAO [7], contributing to failure in meeting synthesis and energy metabolism demands and subsequently reinforcing T cell exhaustion [8] (Fig. 1).

#### Localization

In activated T cells, both mitochondria and the endoplasmic reticulum (ER) are recruited to immune synapses [34, 35]. This close association between mitochondria and the plasma membrane sustains Ca^2+^ release-activated Ca^2+^ (CRAC) channel activity and thus persistent Ca^2+^ influx, enabling Ca^2+^-dependent T cell activation and proliferation [36–39]. Through mitochondria–ER contacts, Akt is rapidly activated by mTORC2, thereby inhibiting Gsk-3β and recruiting hexokinase I (HK-I) to mitochondria. These interactions involve integrated glycolysis and mitochondrial respiration processes [40]. Mitochondria–ER contacts were found to be abundant in CD8^+^ T_M_ cells but rare in T_N_ cells. In addition, suppression of mitochondrial translocation attenuated mitochondrial respiration and subsequently limited T cell activation and function [39].

#### Morphology and structures

Mitochondrial structures are highly dynamic, with this organelle shifting between fusion and fission as a survival mechanism in response to nutrient starvation and stress in T_E_ and T_M_ cells [41, 42]. T_M_ cells are characterized by a long tubular mitochondrial morphology induced via Opa1-dependent fusion, while T_N_ cells exhibit round mitochondria, and T_E_ cells have fragmented mitochondria generated by dynamin-related protein 1 (Drp1)-related fission [39]. Augmenting fusion and inhibiting fission in T_E_ cells elevate cellular respiration because these changes lead to tightly packed cristae and compacted electron transport complexes (ETCs), clearly enhancing T_M_ cell formation and T_M_ cell antitumor function [43–45].

T cell activation and expansion require Drp1-dependent mitochondrial accumulation and mitochondrial fission [46]. Drp1 is translocated to the outer mitochondrial membrane (OMM), where it facilitates aerobic glycolysis, promotes ROS production [47] and induces mitophagy [48]. Drp1 deficiency not only led to reduced T cell infiltration in tumor masses but also increased of the degree of T cell exhaustion [49]. Mitochondrial fusion is regulated by the fusion protein mitofusin 1 (MFN1). In a tumor microenvironment, the exhausted T cell phenotype has been correlated with decreased MFN1 expression and deficient mitochondrial fusion. The exhausted phenotype can be induced through the microRNA-24(miR-24)-MYC-MFN1 axis. In activated T cells under hypoxia, increased miR-24 expression downregulated MYC transcription, thereby reducing the expression of the MYC target MFN1 [50]. Another fusion-associated protein, Opa1, is located on the inner mitochondrial membrane, where it maintains cristae stability and promotes mitochondrial respiration. Opa1-overexpressing mice exhibited longer and more tightly packed cristae in the mitochondria of the heart and other organs, and this mitochondrial structure protected the mice from ischemic damage and muscular atrophy. In exhausted T cells infected with chronic hepatitis B virus, Opa1 expression was significantly downregulated [51].

The aforementioned evidence indicates that T cell subpopulations exhibit different mitochondrial structures that enable their adaptation to perform different functions required for promoting antitumor or anti-infection immunity.

#### Mitochondrial membrane potential

The ΔΨm is generated by proton pumps (ETC complexes I, III and IV). Therefore, the ΔΨm is an intermediate form of energy storage, and the ATP thus maintained is mainly consumed during synthetic processes. The ΔΨm is a driving force for the transportation of charged compounds, some of which are crucial for mitochondrial quantity and dysfunctional mitochondrial elimination. T_SCM_ and T_CM_ subsets were enriched with CD8^+^ T cells exhibiting low ΔΨm, and these cells displayed enhanced in vivo persistence and greater antitumor immune effects than cells with high ΔΨm [52]. Both hyperpolarized and depolarized mitochondria accumulated in exhausted T cells, contributing to T cell metabolic dysfunction and terminal exhaustion. Dysfunctional CD8^+^ tumor-infiltrating lymphocytes (TILs) in clear cell renal cell carcinoma (ccRCC) showed fragmented and hyperpolarized mitochondria and high ROS production [53]. On the other hand, CD8^+^ TILs with depolarized mitochondria expressed higher PD-1 and LAG-3 expression but lower IFN-γ, and they underwent limited population expansion compared to T cells exhibiting lower ΔΨm [32].

### Mitochondrial biogenesis and recycling regulate survival and function in T cells

T cell mitochondrial plasticity, which decisively regulates T cell fate, has been characterized on the basis of not only mitochondrial dynamics, namely fusion and fission, but also of mitochondrial biogenesis and mitophagy. Mitochondrial biogenesis is induced upon T cell receptor (TCR) activation in T cells [54]. Peroxisome proliferator-activated receptor gamma coactivator 1-alpha (PGC-1α) is the master coactivator of mitochondrial biogenesis, serving as an effector molecule in a variety of signaling pathways, such as the mTOR [55], AMPK [56] and MAPK signaling pathways [57], and inhibiting pathways such as the PD-1 signaling pathway. PGC-1α has been shown to activate various transcription factors, such as nuclear respiratory factor 1 and nuclear respiratory factor 2 (NRF1/2). NRF1/2 activated mitochondrial transcription factor A (TFAM), which promote mitochondrial DNA replication and transcription [58]. PD-1 signaling suppressed PGC-1α expression, which inhibited mitochondrial biogenesis, leading to CD8^+^ T cell exhaustion [59]. Notably, it has been reported that exhausted tumor-infiltrating T cells showed progressive PGC-1α loss, which had been induced by continual Akt signaling [60]. Therapeutically, PGC-1α activation favored the differentiation of CD8^+^ T_CM_ cells, and PGC-1α-overexpressing exhausted T cells displayed mitochondrial biogenesis and greater antitumor immunity [61].

Mitophagy, a mitochondrial recycling system, removes damaged or superfluous mitochondria and recycles mitochondrial components. Energy metabolism homeostasis is maintained when mitophagy and mitochondrial biogenesis are balanced, regulating T_M_ cell differentiation and overall T cell survival [62, 63]. Exhausted CD8^+^ T cells exhibited various structural or functional mitochondrial alterations resulting from impaired mitophagy activity [32]. In addition, T cells lacking expression of essential mitophagy genes, such as Atg5, Atg7 and Atg3, accumulated depolarized mitochondria and ROS and acquired T_E_ cell phenotype [64, 65]. Additionally, due to enhanced expression of the proapoptotic molecules pro-caspase-3, caspase-8 and caspase-9 as well as Bim, mitophagy-deficient T cells underwent apoptosis at a higher rate [66, 67]. Mitophagy receptor NIX-mediated mitophagy was critical for the differentiation of effector memory T (T_EM_) cells because it prevented HIF1α accumulation and maintained long-chain fatty acid metabolism [68]. In a hypoxic tumor microenvironment, mitophagy is a metabolic salvage response to counteract overwhelming ROS generation and an impaired FAO process by removing damaged mitochondria, which in turn mitigates T cell death [69, 70].

### Mitochondrial metabolism involves in T cell memory formation or exhaustion

Mitochondria-derived metabolism, including FAO, pyruvate oxidation, the tricarboxylic acid cycle, glutaminolysis and one-carbon metabolism, provides not only energy and biosynthetic precursors through aerobic respiration but also intermediates such as cell signaling messengers and antioxidants. Recently, enhanced mitochondrial spare respiratory capacity (SRC) was shown to be preferentially associated with T_CM_ cells, which show longer-term survival [71, 72]. In preclinical models, glycolysis-dominant T cells were short-lived after adoptive transfer and showed defective antitumor immune effects, while inhibiting glycolysis drove CD8^+^ T_M_ cell differentiation and enhanced antitumor function [73].

#### OXPHOS

T_N_ and T_M_ T cells are generally considered to undergo mitochondrial FAO and maintain SRC to ensure their long-term survival and persistence advantages [74]. During T_N_ cell activation, both the aerobic glycolysis and OXPHOS rates are accelerated, and aerobic glycolysis is induced by the costimulation of TCRs and CD28 receptors to support T_E_ cell differentiation and function, such as cytokine production [13, 44]. A recent study showed that CAR-T cells exhibited enhanced mitochondrial biogenesis and OXPHOS, contributing to T_M_ cell differentiation and persistence [75]. Upon T cell exhaustion during chronic infection or tumorigenesis, glycolysis and OXPHOS were suppressed by the PD-1-PD-L1 pathway, which reduced glucose uptake and repressed expression of PGC-1-α [59, 60], and this metabolic dysregulation led to lost proliferative capacity, referred to as the “terminally exhausted” phenotype [59, 76, 77]. In addition, terminally exhausted T cells caused by chronic antigen stimulation showed decreased mitochondrial respiration. To compensate, glycolysis was accelerated, and spare glycolytic capacity was markedly reduced to maintain bioenergetic homeostasis [8]. Consistent with these findings, single-cell transcriptome analyses have revealed an enriched glycolytic gene signature in terminally exhausted T cell populations compared to either early exhausted or T_E_ cells in mouse or human tumors [8, 13]. Inhibition of mitochondrial OXPHOS by ETC complex inhibitors limited T cell self-renewal capacity and upregulated the expression of exhaustion-associated genes in T cells [8, 77].

**Fig. 2:**
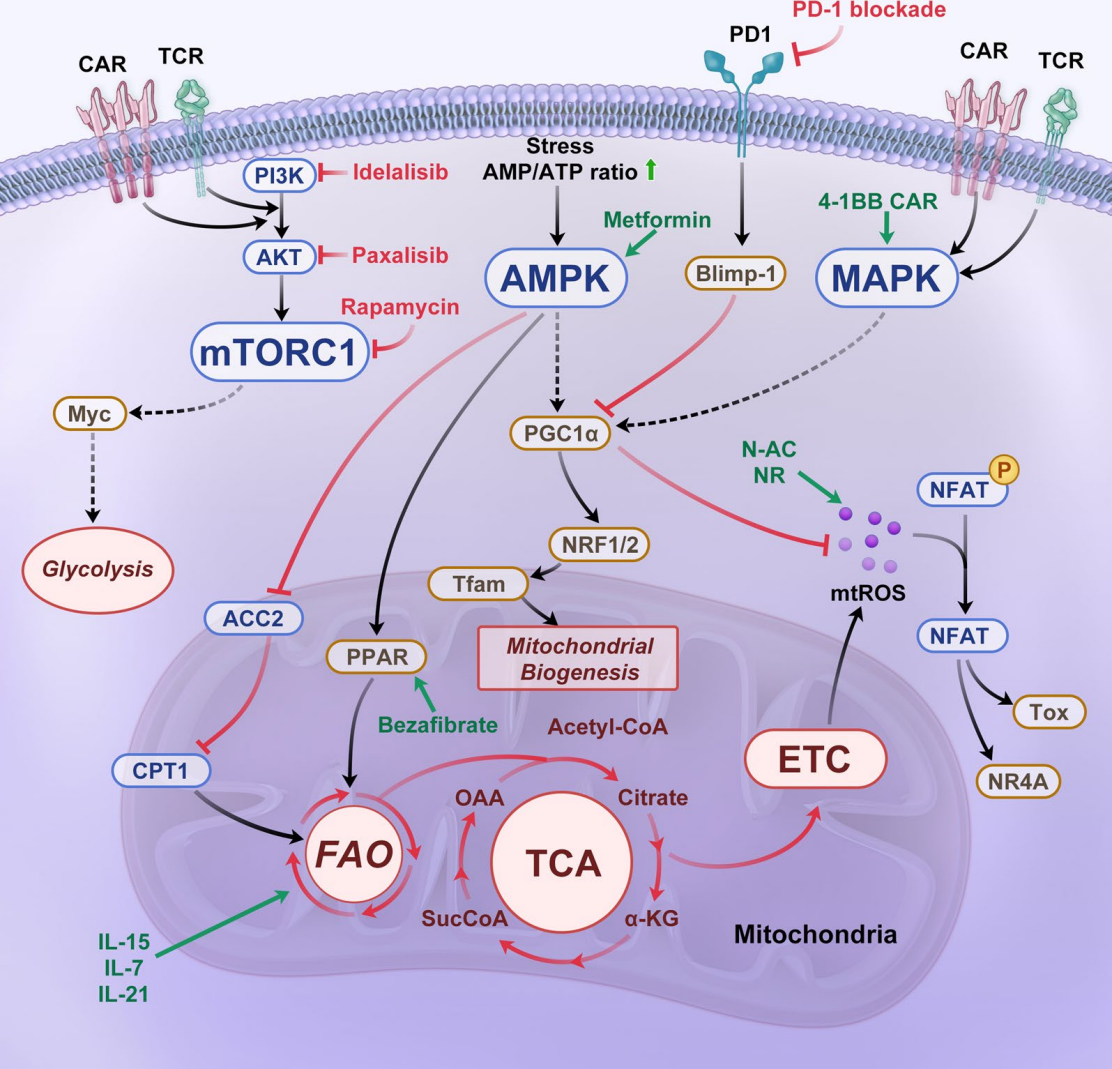
Cell signaling associated with mitochondrial metabolism and strategies to counteract CAR-T cell exhaustion. A variety of signaling pathways, such as the mTOR, AMPK, and MAPK signaling pathways, and inhibiting pathways, such as the PD-1 signaling pathway, are involved in T cell activation and mitochondrial metabolism. When activated, metabolic switching mediated by the PI3K–PKB (AKT)–mTOR pathway supports the differentiation of effector T cells (T_E_) cells. The AMPK pathway drives long-chain fatty acid oxidation (FAO) and mitochondrial biogenesis through phosphorylation of ACC2 and PGC-1α activity. PGC-1α activates NRF1/2, which activate Tfam. Tfam drives the transcription and replication of mitochondrial DNA (mtDNA). In addition, 4-1BB signaling upregulates PGC-1α expression through stimulation of the p38-MAPK pathway, resulting in mitochondrial fusion and biogenesis. Increased PD-1 inhibitory receptor expression induces Blimp-1 expression, a critical exhaustion-related transcription factor, to exacerbate NFAT activity. NFAT activates the transcription factor TOX, which is associated with cell exhaustion and suppresses the expression of transcription factor Tcf7, which is related to oxidative phosphorylation (OXPHOS)

A number of activated transcription factors have been shown to be critical regulators of T cell metabolism, such as mTOR, AMPK and c-Myc (Fig. 2). mTOR and AMPK, complementary sensors of intracellular energy status, are the central controllers of biosynthesis and catabolism [78, 79]. mTORC1 activity sustains high levels of aerobic glycolysis in T_E_ cells, and inhibition of mTORC1 activity reduces glucose uptake and lactate production in IL-2-maintained T_E_ cells [80]. In contrast, mTORC2 is not required for T cell effector function. CD8^+^ T_E_ cells with reduced mTORC2 activity showed robust effector function while simultaneously demonstrating an increased rate of differentiation into T_M_ cells. When IL-2 levels were maintained, T cells with diminished mTORC2 activity continued to exhibit high SRC and FAO levels, which promoted T_M_ cell development [81]. AMPK has been shown to drive long-chain FAO and mitochondrial biogenesis by promoting the phosphorylation of acetyl-CoA carboxylase (ACC) 2 and activation of PGC-1α [82] to promote T_M_ cell development. In addition, the exhausted T cell phenotype has been related to Myc, which drives a transcription program that reduces glucose catabolism and glutamine oxidation after T cell activation [83, 84].

#### Glutaminolysis

Glutaminolysis is a vital pleiotropic process that leads to glutamine catabolism, producing ATP and alpha-ketoglutarate (α-KG), which are biosynthetic intermediates [85]. Within mitochondria, glutamine is converted into glutamate by glutaminase (GLS) and then subsequently converted to α-KG, which participates either in the tricarboxylic acid (TCA) cycle to support OXPHOS [86] or is exported out of mitochondria to participate in fatty acid biosynthesis and contribute to redox balance [87]. In activated immune cells, glutamine metabolism is accelerated to support T cell expansion, differentiation and function. Generally, glutamine depletion inhibits T cell proliferation and reduces cytokine production (mainly IL-2 and IFN-γ) in activated T cells [88]. Interestingly, GLS1-knockout CD4(+) T cells exhibited an increase in the proportion of T helper 1 (TH1) cells, which showed excessive effector function and an increase in exhaustion signatures, but in cytotoxic CD8+ T cells in culture with limited glutamine or treated with a glutamine metabolism inhibitor prevented exhaustion, promoted memory-like T cell differentiation and tumor elimination in vivo [89, 90].

#### One-carbon metabolism

One-carbon metabolism functions simultaneously in mitochondria and the cytosol. Mass spectrometry (MS) analyses have revealed that mitochondrial one-carbon metabolism is one of the most highly induced pathways during T cell activation, suggesting that one-carbon metabolism is critical for T cell activation [91]. Single-carbon units are derived from serine through methyltransferase SHMT2 action in mitochondria [92] and are accepted by tetrahydrofolate [93]. One-carbon metabolism was shown to regulate T cell proliferation and survival, as indicated by SHMT2 knock out impairing respiratory chain enzyme activity via folate-dependent transfer RNA (tRNA) methylation and reducing T cell viability [91]. Enhanced one-carbon metabolism contributed to purine/thymidine and glutathione synthesis [94]. Importantly, purine/thymidine enables rapid T cell expansion, and the glutathione prevents ROS accumulation.

These data suggested that among subsets of T cells in typically heterogeneous T cell populations exhibit large variation in FAO, glycolysis, glutaminolysis, OXPHOS and one-carbon metabolism. Although described independently, these metabolic pathways are interconnected through mutual intermediates or crosstalk with metabolites or signaling pathway components, regulating T cell survival, proliferation, functional differentiation and metabolic reprogramming.

### Mitochondrial ROS exhibit bidirectional functions in T cells

Generally, TCR signaling increases the intracellular production of ROS, such as superoxide (O^2−^) and hydrogen peroxide (H_2_O_2_). ROS are mainly generated from mitochondria [95] and membrane-bound NADPH oxidases [96]. Mitochondria-derived ROS, postulated to participate in the TCR signaling pathway, are critical for IL-2 production via NF-κB signaling and nuclear factor of activated T cell (NFAT) activation [97]. A previous study found that counteracting ROS action with antioxidants reduced the expansion of T cells during primary viral infection [98]. These results highlighted the role of ROS in T cell activation. Notably, ROS overload can induce T cell dysfunction, DNA damage, apoptosis and necrosis [99].

Single-cell RNA-seq data have revealed significant enrichment of oxidative stress-related genes in terminally exhausted T cells [8]. Oxidative stress caused by ROS accumulation is sufficient to impair T cell proliferation and self-renewal, resulting in exhaustion [8]. An increase in intracellular calcium after continuous antigen stimulation increases mitochondrial ROS, followed by the upregulated expression of inhibitory receptors, including PD-1, TIGIT, LAG-3 and TIM-3 [8], and progressively decreased TCF1 expression, which favors the formation of CD8 + T_M_ cells [97, 100]. PGC-1α, as mentioned above, is a transcription coactivator that not only coordinates mitochondrial biogenesis but also attenuates oxidative stress by promoting the expression of antioxidant enzymes [101]. Blimp-1 represses PGC-1α expression and thus is critical for excessive ROS generation in exhausted T cells [102]. Notably, short interfering RNA (siRNA) knockdown of Blimp-1 expression or overexpression of PGC-1α has been shown to reverse metabolic dysregulation and attenuate oxidative stress and functional defects in exhausted T cells [103, 104].

Reprogramming of mitochondrial dynamics, mitochondrial biogenesis and recycling, mitochondrial metabolism and changes to the redox balance during T cell fate, as discussed above, are all closely associated with TCR signaling. In this respect, CAR-T cells, which are considered excellent imitators, share similarity with conventional T cells. Enhanced mitochondrial biogenesis, a higher SRC and accelerated FAO increase the proportion of T_M_ cells in the total CAR-T cell population and prevent cell exhaustion [9]. However, in contrast to conventional T cells, tonic signaling makes CAR-T cells more prone to exhaustion even before encountering antigen. During manufacturing and expansion of the CAR-T cells in vitro, T cells isolated from patients are stimulated with anti-CD3/28 beads or antibody, followed by delivering CARs to the T cells. In this process, exhaustion is, overall, a greater threat to CAR-T cells than to T cells and negatively affects CAR-T cell therapy. CAR-T cells show high metabolic activity, especially after CD28 costimulation, which enhances anabolic metabolism to promote proliferation and differentiation into T_E_ cells. In contrast to CAR-T cell activity with CD28 costimulation, 4-1BB CAR-T cells displayed higher mitochondrial metabolic activity [9]. Thus far, the degree to which mitochondrial-related cellular processes in CAR-T cells affect survival, differentiation and exhaustion and the underlying mechanisms of these effects have not yet been investigated in CAR-T cells.

## Strategies to counteract CAR-T exhaustion by targeting mitochondrial metabolism

### Costimulatory domain

The most widely used costimulatory endodomains in the CAR molecule are derived from the CD28 or 4-1BB genes. CD28 stimulation activates the PI3K-Akt pathway, leading to enhanced aerobic glycolysis mediated by downstream pathways [105]. GD2 CAR-based CD28 costimulation promotes the expansion of CAR-T cells but induces their rapid exhaustion, low cytokine production and short persistence in vivo [2]. In contrast, endogenous 4-1BB signaling upregulates PGC-1α through stimulation of the p38-MAPK pathway, resulting in mitochondrial fusion and biogenesis and substantially increasing T cell respiratory capacity. This outcome enhances the antitumor immunity and long-term survival of T cells [10, 106]. The clinical efficacy of CAR-T cells with a 4-1BB costimulatory domain is superior to that of CD28z-stimulated CAR-T cells in chronic lymphocytic leukemia (CLL) patients [107, 108] (Fig. 2).

### Cytokines

Culturing methods, including the cytokine composition in the medium, often impact the potency of the CAR-T cell product. To date, cytokines, including IL-2, IL-7, IL-15 and IL-21, have been thoroughly investigated for manufacturing CAR-T cells (Fig. 2). IL-2 is a T cell growth factor that promotes glycolysis and subsequent differentiation of CD8^+^ T cells in T_E_ cells [109, 110]. CAR-T cells are usually expanded by IL-2, which increases T_E_ differentiation by activating the Akt-mTOR signaling pathway. However, Huang et al. found that IL-2–IL-2R signaling resulted in aryl hydrocarbon receptor activation, leading to CD8^+^ T cell exhaustion in tumor environments [111]. In contrast, IL-7 and IL-15 have been implicated in the maintenance of T_M_ cells [71, 112, 113]. IL-7 enhances CD8^+^ T_M_ cell formation by inducing the expression of glycerol channels and increasing triglyceride (TAG) synthesis [114]. IL-15 increases the SRC and oxidative metabolism rate by enhancing mitochondrial biogenesis and CPT1a expression [71]. CAR-T cells expanded upon exposure to IL15 (CAR-T/IL15) exhibited a T_SCM_ phenotype, which was closely associated with decreased mTORC1 activity and reduced expression of glycolytic enzymes [6]. Moreover, IL-21 modulated the induction of T_CM_ cells and acquisition of the exhaustion phenotype, increasing antitumor efficacy in an FAO-dependent manner [115]. Strategies combining these cytokine compositions in cell culture medium show promise for increasing T_SCM_ or T_CM_ populations and boosting antitumor efficacy [116].

### Metabolic signaling regulators

Manipulation of metabolism-regulating signaling and mitochondrial activation has been suggested to improve CAR-T cell fate and reverse exhaustion (Table 1 and Fig. 2). Sustained PI3K–Akt–mTOR pathway activation caused by persistent stimulation with beads or IL2 or tonic signaling leads to terminal differentiation of CAR-T cells and impairs antitumor immunity. Blocking the PI3K-AKT-mTOR signaling pathway preserves the T_EM_ population [81, 117, 118]. PI3K inhibitors (LY294002, IC87114, idelalisib and TGR-1202) optimally maintained CAR-T cells in a less differentiated state in vitro without impairing their activation, leading to significantly longer CAR-T cell persistence and enhanced antitumor capabilities in vivo [119–121]. However, the positive effect of PI3K inhibitors on CAR-T cell expansion is conditional. Blocking both the PI3K gamma and delta catalytic subunits reduced effector cytokine production after antigen rechallenge and decreased CAR-T cell persistence in vivo. Only CAR-T cells treated with either PI3Kγ or PI3Kδ inhibitors acquired a T_CM_ phenotype and showed enhanced antitumor activity in vivo [121]. AKT inhibition had relatively little impact on glycolysis while promoting the accumulation of both long-chain and polyunsaturated fatty acids in T cells, accelerating FAO and augmenting mitochondrial SRC [122]. Since mTORC2 is an Akt activator, functional mTORC2 deficiency enhanced CD8^+^ T_M_ cell formation by accelerating FAO and mitochondrial metabolism [123]. After pretreatment with AKT inhibitors, CAR-T cells preserved memory-like characteristics, increased cytokine production and greater degrees of antitumor efficacy and expansion in vivo [124–126]. These properties may be associated with repressed expression of apoptosis-associated genes, including BAX, TRAIL and BAD, and upregulated expression of persistence- and trafficking-related genes, such as IL7R, S1PR1 and KLF2 [127]. PD-1 signaling has been shown to decrease glucose metabolism and inhibit AKT activation by blocking PI3K activation [128].

**Table 1:** Metabolic signaling regulators that improve CAR-T cell destiny and reverse exhaustion

Target	Compounds	FDA approval	Model	Outcome	References
PI3K	LY294002 IC87114	No	1. MOLM-13-CD19 cell line 2. NSG mice AML model	Increasing T_N_ and T_CM_ populations of CD33 CAR-T in vivo Improving CAR-T cell persistence and reducing tumor burden in vivo	[119]
PDK Glycolysis	Dichloroacetate	Yes	1. Hepatocellular carcinoma (HCC) cell lines 2. HCC-bearing mice model	Increasing TN and TCM percentages Reducing lactate-mediated immunosuppression	[168]
mTOR	Rapamycin	Yes	Acute lymphocytic choriomeningitis virus infection mice model	Enhancing the formation of memory CD8 T cells during the naïve to effector T cell differentiation phase and the effector to memory transition phase in vivo Improving the functional qualities of the memory CD8 T cells in vivo	[117]
Akt	A-443654	No	LCMV-infected mice model	Rescuing short-lived effector cells from deletion due to sustained Akt activation Enhancing P14 CD8 T_EM_ cells in vivo	[118]
Akt	Akti-1/2	No	1. CD19 + lymphoid leukemic cell line 2. CD19 + tumor-bearing immunodeficient mice model	Preventing CAR-T cell differentiation Increasing cytokine production and cytotoxicity Exhibiting greater anti-tumor efficacy and expansion in vivo	[124]
BATF c-Myc	JQ1	No	1. K562 cell line, CD19 + leukemia cell line NALM-6,melanoma cell line A375 2. ALL or melanoma-bearing NSG mice model	Promoting the maintenance of TSCM and TCM phenotypes, persistence and antitumor effects of CAR-T cells in vivo	[169]
PI3Kγ PI3Kδ	IPI-549 CAL-101 TGR-1202	No	1. B16F10 (H-2b) melanoma cell line 2. K562-mesothelinexpressing cell line 3. pmel-1 TCR-transgenic mice model	Sole blockade of PI3Kγ or PI3Kδ, but not dual inhibition, induces a naive/stem memory-like profile and enhances in vivo antitumor immunity of infused CD8^+^ T cells PI3Kδ inhibition improves cytotoxicity of Meso-CAR T cells	[121]
PI3Kδ	Idelalisib	Yes	1. PBMCs fromconsenting DLBCL patients and healthy controls 2. NSG mice model 3. B6 SJL mice lymphoma model	Enhancing the expansion and functionality of CAR-T cells in vitro Enhancing DLBCL patient T cells persistence in vivo Enhancing anti-tumor activity of OT-T cells in a murine lymphoma model	[120]
PGC-1α PPAR-α	Bezafibrate Fenofibrate	Yes	1. Melanoma mice model 2. Skin sarcoma BALB/c mice model	Promoting FAO Improving CD8^+^ TIL functions Synergizing with PD-1 blockade	[133, 136, 170]

Inhibition of mTORC1 by rapamycin or the AMPK activator metformin promoted memory CD8^+^ T cell formation by accelerating FAO and overall OXPHOS rates [81, 122]. An inhibitor of the MAPK signaling pathway, MEK1/2i, induced the acquisition of a T_SCM_-like phenotype by suppressing cyclin D1 expression, postponing cell cycle progression and enhancing mitochondrial biogenesis while maintaining normal TCR-mediated activation through the ERK1/2–cyclin D1–PGC-1α–SIRT3–FAO pathway [129]. Notch signaling contributes to T_SCM_-like differentiation of CAR-T cells by enhancing mitochondrial biogenesis and telomere elongation via its downstream effector FOXM1 and thus enhances CAR-T cell antitumor potential [130]. Other signaling regulators, such as the tyrosine kinase inhibitor dasatinib, have also been considered optimal candidates for reversing CAR-T cell-terminated differentiation and exhaustion during ex vivo expansion as well as in vivo persistence [131, 132].

Direct mitochondrial-targeting agonists also show promised for enhancing CAR-T cell functions and reversing T cell exhaustion [133, 134]. Agonists of PGC-1α or PPAR-α (bezafibrate and fenofibrate, respectively) enhanced proliferation and increased the survival of T_N_ cells and increased CD8^+^ T cell function, which was supported by increased mitochondrial fatty acid catabolism and subsequent accelerated OXPHOS, as well as increased glycolysis [135, 136].

### Redox homeostasis

Treatment with mitochondria-targeted (MT) antioxidants may be a new strategy to prevent cytotoxic T cell exhaustion. Antioxidant treatment restores the metabolic function and self-renewal capacity of exhausted T cells during continual antigen stimulation both in vitro and in vivo. Two MT antioxidants, mitoquinone and piperidine-nitroxide MitoTEMPO, attenuate oxidative stress induced by defective ΔΨm depolarization and excessive ROS levels in exhausted CD8 + T cells, significantly enhancing cell viability and antiviral function [77]. N-acetylcysteine (N-AC) supplementation of chronically stimulated T cells increased the synthesis of glutathione, a critical antioxidant, which further reduced ROS accumulation and increased the oxygen consumption rate (OCR). N-AC-treated T cells undergo a phenotypic transition from terminally exhaustion to progenitor exhaustion, which is identified by one-half of cells expressing both TCF-1 and TOX (Fig. 2). The mechanism by which terminal T cell exhaustion is thus prevented may involve attenuation of Ca^2+^-driven, ROS-dependent NFAT activity [137]. In addition, supplementation with nicotinamide ribose (NR), a precursor of another important antioxidant, reduced nicotinamide adenine dinucleotide (NADH), which prevented the decline in mitochondrial fitness (Fig. 2). After either intratumoral injection or oral administration, NR significantly attenuated mtROS levels and increased the effector cytokine production mediated by antigen stimulation of CD8^+^ T cells [32]. Regarding CAR-T cell therapy, Ligtenberg et al. presented a strategy to coexpress catalase with a CAR construct (CAR-CAT) in adoptive T cells to neutralize H_2_O_2_ and thus increase cellular antioxidative capacity. CAR-CAT-T cells exhibited upregulated intracellular catalase expression levels and showed reduced ROS accumulation in the primary or activated state while maintaining viability and antitumor function under oxidative stress [138].

Importantly, these findings indicate that genetic or pharmacological disruption to T cell metabolism may mediate T cell differentiation preferences and determine T cell fate. To enhance antitumor efficacy, the metabolic state of CAR-T cells can be reprogrammed by changing the CAR construct or culture conditions to balance glycolysis and FAO, although further testing is needed to fully validate this suggestion.

## New insights: metabolic analysis of T cells and CAR-T cells based on single-cell techniques

Metabolic enzymes and intermediates exert indispensable regulatory effects on CAR-T cell proliferation, differentiation, functional activation and persistence. Important mechanisms of metabolic regulation, including CAR-T cell metabolic status reprogramming to meet the needs for both energy and biosynthesis during rapid expansion, and how these mechanisms balance divergent metabolic pathways to direct the formation of long-lived T_M_ or short-lived T_E_ cells are worthy of further investigation.

**Fig. 3:**
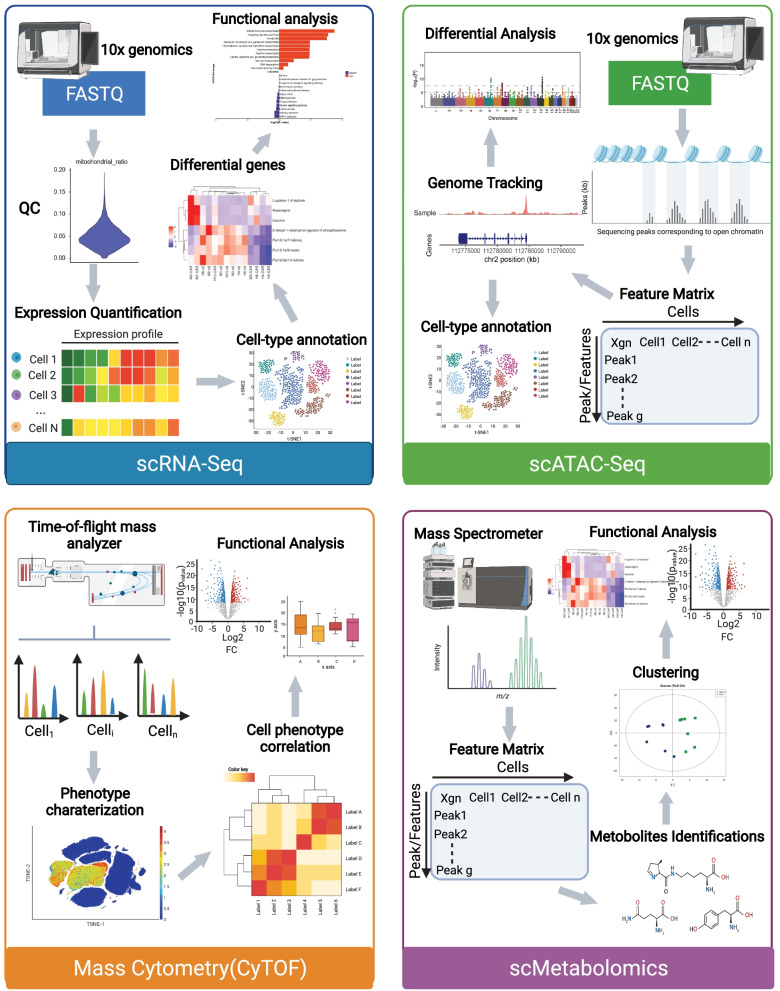
Metabolic analysis of T and CAR-T cells based on single-cell techniques

To date, cellular metabolic analyses have been based mostly on bulk assays, mainly involving quantification of metabolic intermediates, products and substrates (e.g., MS and extracellular flux analysis). However, because T cells exhibit tremendous heterogeneity, sophisticated measurement technologies are urgently needed to characterize metabolic profiles (including the expression levels of metabolic genes, the activity of metabolic enzymes and the abundance of metabolites) at single-cell resolution (Table 2) (Fig. 3).

**Table 2:** Metabolic analysis of T cells and CAR-T cells based on single-cell techniques

Type	Method	Cell types	Cell source	Main conclusion	References
Single-cell RNA-seq	10X Genomics	CAR-T cells	5 healthy donors	41BB CAR-T cells had increased markers associated with CD8 central memory T cells and favored fatty acid metabolism	[141]
	1. Bulk-seq 2. 10X Genomics	CAR-T cells	24 patients with LBCL, 137,326 residual cells	Heterogeneity of CAR T cell infusion products contributes to variation in efficacy and toxicity after axi-cel therapy	[171]
	scTCR/transcriptome by 10X Genomics	peripheral blood lymphocytes after TIL infusion	1 patient with metastatic colorectal cancer	After ACT, the TILs gene expression patterns changed, but IL7R, ITGB1, KLF2 and ZNF683 remained expressed in the persistent clonotypes, compared to the non-persistent clonotypes	[161]
Single-cell Proteome	1. CyTOF 2. MIBI-TOF	CD8 T cells	1. Tumor tissue (n = 6) and healthy adjacent tissue from the same patients (n = 6) 2. Unrelated healthy donor PBMCs (n = 5) and lymph node biopsies (n = 3)	1. Three check points were identified of metabolic switching of naive human CD8^+^ T cells 2. Single-cell metabolic regulome profiling(scMEP) metabolic states associated clearly with distinct immunological phenotypes	[148]
	Mass Cytometry	CD8 T cells CAR-T cells	1. Naive or LMV infected WT C57BL/6 mice 2. 2 advanced NHL patients receiving axi-cel therapy	A distinct metabolic state in early-activate T cells (most abundant 5 days post-infection) characterized by maximal expression of glycolytic and oxidative metabolic proteins	[150]
	Met-Flow	Human PBMCs	12 donors, 150,000 cells per leukocyte population	1. Metabolic remodeling occurs during T cell activation 2. T cell memory subsets show differential metabolic phenotypes; The TCM and TEM populations both expressed higher levels of ACAC, PRDX2, and CPT1A, in contrast to naive and TTEMRA subsets	[172]
Multiplexed single-cell approaches	Ins-seq	Monocytes and macrophages	7648 cells from MCA205 tumor-bearing mice	1. Arginase 1-expressing cells within tumor is a metabolic immune signature of suppressive activity 2.A novel group of Arg1 + Trem2 + regulatory myloid(Mreg) cells was found and cellular markers, metabolic activity and associated pathways were defined	[160]
	1. 10X Genomics 2. Compass	Pathogenic and non-pathogenic Th17 cells	C57BL/6 wild-type mice	1. Pathogenic Th17 maintain higher aerobic glycolysis and TCA activity, whereas non-pathogenic Th17 oxidize fatty acids to produce ATP 2. Th17 pathogenicity was associated with arginine and downstream polyamine metabolism with enhanced polyamine-related enzyme expression in pathogenic Th17 and suppressed levels in Treg cells	[173]
	1. scRNA-seq 2. Mass cytometry 3. scATAC-seq	Human PBMCs	1. Young/Aged healthy adults 2. Young/Aged COVID-19 patients 3. Young/Aged COVID-19 patients recovered	Immune cell landscape was reprogrammed with age and was characterized by T cell polarization from naive and memory cells to effector, cytotoxic, exhausted and regulatory cells, along with other immune cells	[163]

### Single-cell transcriptome analysis

Single-cell RNA sequencing (scRNA-seq) permits comparisons of individual cell transcriptomes and understanding cellular phenotypes as ascertained by unbiased clustering, which is a measurement suited for discovering novel cell types, dissecting cell fate decisions (differentiation), or characterizing highly heterogeneous T cell populations [29, 139]. Based on scRNA-seq and TCR-seq, CD8+ T cells previously defined as exhausted were, in fact, proliferating clones of differentiating cells in the human tumor microenvironment [29]. A series of transcriptional atlases associated with CAR-T cell exhaustion [140], treatment efficacy and induced toxicity [141] have been generated through scRNA-seq analyses of CAR-T cell products and treatment progression [142]. These studies have provided unprecedented opportunities to understand the heterogeneity of the cell subsets and molecular features of the CAR-T cell population and revealed the factors correlated with clinical outcomes to guide patient management.

Single-cell metabolic profiling based on scRNA-seq datasets can be analyzed to demonstrate the behavior of metabolic genes, such as the glycolytic activity gene Glut1, FAO components CPT1 and CPT2, and oxidative stress response factor NRF2 [143, 144]. By comparing single-cell profiles of CAR-T cells with different intracellular domains at rest and activation, 41BB CAR-T cells have enriched genes associated with CD8 T_CM_ cells and favored fatty acid metabolism at rest, which is likely to be a key driver of prolonged persistence [141]. CRISPR–Cas9 genome-wide screening combined with scRNA-seq has emerged as a powerful approach to identify essential regulators of CAR-T cells and inhibit exhaustion responses [145, 146]. To date, scRNA-seq has been performed to characterize the metabolism of CAR-T cell therapies and to clinically monitor CAR-T cell therapies. However, future studies should be possible to interrogate metabolic molecular targets toward clinical benefit in CAR T therapies.

### Single-cell proteome analysis

The utility of antibody-based proteomic platforms is able to quantify over 130 isotopes simultaneously, including surface markers, rate-limiting enzymes, metabolic transporters and epigenetic modifications of individual cells [147]. Changes in metabolic activity in T cells observed at single-cell resolution through protein quantification have a high degree of consistency with the changes measured by extracellular flux analysis [148, 149]. Furthermore, in-depth single-cell-based proteome analysis of T cells can identify distinct phases of T cell remodeling as well as their relationship to transitions between cellular states, which could not be achieved by bulk analysis.

Remarkable cell type-specific metabolic diversification is revealed within the human immune system [149]; for example, early activated T cells exhibit maximal glycolytic activity and increased mitochondrial mass and activity [150]. According to a recent study, proteins that regulate metabolic pathway activity were quantified in human cytotoxic T cells based on high-dimensional antibody-based technologies. It defines three inflection points during metabolic remodeling of naïve human CD8 + T_N_ cell activation: accelerated upregulation of metabolic proteins, RNA synthesis initiation together with cellular stress response activation, and stabilized metabolic protein expression accompanied by peak translational activity [148]. The study also revealed that cytotoxic T cells expressing the exhaustion-associated molecules CD39 and PD-1 diverged into two subsets in human tumors. Of these, decreased metabolism (i.e., reduced GLS, GOT2, PFK2, ATP5A, CS, CytC and PGC1a_p) was proposed to result in terminally exhausted T cells based on low expression of TCF1 [148]. Single-cell proteomes can further reveal the metabolic dynamics of CAR-T cells. CAR-T cell products before infusion exhibited peak oxidative and glycolytic marker expression. Thereafter, most metabolic proteins decreased by 7, 14, 21, 28 and 90 days after CAR-T cell infusion [150]. These studies suggest that T cell capacities could be defined via an additional dimension of the metabolic state.

In addition to cytometry-based approaches for protein-level measurements, approaches can include assessment of nonprotein markers of cellular phenotypes, such as the mitochondrial potential dye MitoTracker, a fluorescent derivative of glucose 2-NBDG. This technique may be helpful in understanding multiple metabolic parameters affected in the immune system. However, the sensitivity of these methods is still limited to only tens to hundreds of protein markers per cell. Spectral flow cytometry, capillary electrophoresis–electrospray ionization-MS (CE-ESI–MS) and nanoscale liquid chromatography coupled to MS (nano-LC–MS)-based platforms are used in single-cell proteome analysis and meet the sensitivity requirements to detect hundreds to thousands of proteins [151–153]. These developing MS techniques herald a new era of high-throughput single-cell analysis by enabling label-free protein quantification.

### Single-cell metabolomic analysis

Metabolites are small molecules, usually less than 1.5 kD, and include lipids, sugars, glycolytic metabolites and phosphate compounds [154]. Metabolic flux is often reported as a reaction rate of a metabolic pathway in a steady state, reflecting the interactions of various processes such as transcription, translation and enzyme activity and metabolite concentration [155].

Single-cell metabolomics can explain the molecular heterogeneity and dynamic states of mechanisms within complex immune tissues and their cellular microenvironments. However, due to extremely low levels of metabolites in a single cell and the lack of a high-throughput cell-processing technique to extract metabolites, single-cell metabolomics technology is currently not sufficiently mature for large-scale applications. The instability of metabolites can be partially resolved by integrating metabolomics with other data, such as transcriptomic and proteomic data, which not only can be used in cross-validation but also can be used, more importantly, to analyze metabolic changes in different cell types and stages of cell development through pseudotemporal analysis in immunometabolism research.

### Integration of multiplexed single-cell approaches

Cell signaling and epigenetic, transcriptional and metabolic pathways are integrated to regulate cell function. By combining high-precision genome-sequencing approaches, we obtained highly reliable cell lineage trees that revealed cell genetic variants, and these variants can be measured as cell clonal markers to track cell dynamics.

Several newly developed multiplex single-cell techniques are currently under investigation. Proximity extension assays (PEAs) provide details of protein and transcript abundance in the same single cell at the same time on the basis of oligonucleotide-labeled antibody measurements [156, 157]. Based on the oligonucleotide-labeled antibody method, a high-throughput scRNA-seq method called cellular indexing of transcriptomes and epitopes by sequencing (CITE-seq) has been established. As a multimodal data analysis platform, CITE-seq integrates surface cell protein and transcriptome measurements and provides more detailed characterization of cellular phenotypes (natural killer [NK] cell, T cell, etc.) than transcriptome measurements alone [158, 159]. Insertion sequencing (IN-seq), another integrated technology for massive and parallel recording of scRNA-seq data and intracellular protein activity, can be broadly applied for elucidating integrated transcriptional and intracellular maps and defining new immune subsets [160]. By sequencing TCR RNA transcripts and tracing them in the study of clonal dynamics, clonotypes and phenotypes can be directly compared.

On the basis of scRNA-seq and scTCR-seq, two different TIL populations with distinctive gene expression profiles have been found to be associated with the persistence of TILs in a patient [161, 162]. Wenru Su et al. applied scRNA-seq, cytometry by time of flight (CyTOF), a single-cell assay for transposase-accessible chromatin sequencing (scATAC-seq) and single-cell paired T/B-cell receptor sequencing (scTCR/BCR-seq) to compare the properties of peripheral blood mononuclear cells (PBMCs) in young and old adults with SARS-CoV-2 infection. The study revealed that T cells showed decreased diversity and increased phenotype switching, from T_N_ and T_M_ cells to T_E_, exhausted and Tregs during aging [163]. By multiplexing these single-cell techniques, data in addition to those of the transcriptome can be acquired, and particular advances in genomic, chromatin, methylation and proteomic assays can be used to define new cell populations and explore their specific functions in the immune system.

Currently, single-cell multiplex approaches provide unique opportunities to determine common principles of immunometabolic heterogeneity in the immune system. With technological development, the spatial omics of single cells in an original tissue can enable the determination of cell–cell interactions and microenvironmental influences on the cellular state. Moreover, computational models can be optimized to predict cellular phenotypes more accurately. Given certain caveats, the information obtained through single-cell research has introduced the possibility of improving CAR-T therapies against cancers by manipulating the expression of key metabolism-related genes, proteins or metabolites, leading to CAR-T cell products better suited for immunotherapy.

In addition to conventional mitochondrial techniques, such as a Seahorse analysis and live-mitochondria staining, novel techniques based on mitochondria analysis are currently under investigation. Mitochondrial mutation analysis combined with single-cell genomics allows lineage tracing [164]. Lareau et al. [165] developed a mtscATAC-seq method that combines high-confidence mtDNA mutations with the concomitant accessible chromatin profile. mtscATAC-seq enables researchers to link epigenomic variability to subclonal evolution and infer the dynamics of differentiating hematopoietic cells. Furthermore, new mitochondria-related techniques can be used to discover intercellular interactions and reveal pathological mechanisms [166]. By using field-emission scanning electron microscopy and mitochondrial transfer tracing, the nanotube-mediated transfer of mitochondria from immune cells to cancer cells has been characterized; in summary, this transfer enhanced the metabolism of cancer cells and depleted that of immune cells [167].

## Conclusion

Recent studies have indicated that metabolic pathways not only provide building blocks for T-and CAR-T cell during fate determination but also exert a significant influence on the outcomes of CAR-T cell therapy. Indeed, immunometabolism as a systematic science would be a promising field for future research to better understand regulatory mechanisms of CAR-T cells. Understanding the spatiotemporal aspect of CAR-T cell metabolism requires the further development of more novel technologies in the effort to improve CAR-T cell function and treatment outcomes. “Omics” technologies pertain the entire set of molecules present at the cellular level or within an organism under a specific set of conditions. Combining those technologies with single-cell technology like microfluidics, high-resolution imaging etc., metabolic research has entered the era of multi-omics at single-cell level.

## Data Availability

Not applicable.

## Abbreviations

CAR-T: Chimeric antigen receptor T cell
ACT: Adoptive cell therapy
ROS: Reactive oxygen species
OXPHOS: Oxidative phosphorylation
T_CM_: Central memory T cells
FAO: Fatty acid oxidation
T_N_: Naïve T cell
T_M_: Memory T cell
T_E_: Effector T cell
Treg: Regulatory T cell
scFV: Single-chain variable fragment
CAR-T_SCM_: Memory stem cell-like CAR-T cell
mtROS: Mitochondrial reactive oxygen species
ER: Endoplasmic reticulum
CRAC: Ca^2^^+^ release-activated Ca^2^^+^ channel
HK-I: Hexokinase I
Drp1: Dynamin-related protein 1
ETC: Electron transport complexes
OMM: Outer mitochondrial membrane
MFN1: Mitofusin 1
TILs: Tumor-infiltrating lymphocytes
ccRCC: Clear cell renal cell carcinoma
TCR: T cell receptor
PGC-1α: Peroxisome proliferator-activated receptor gamma coactivator 1-alpha
NRF1/2: Nuclear respiratory factor 1/2
TFAM: Mitochondrial transcription factor A
T_EM_: Effector memory T cell
SRC: Spare respiratory capacity
ACC: Acetyl-CoA carboxylase
α-KG: Alpha-ketoglutarate
GLS: Glutaminase
TH1: T helper 1 cell
MS: Mass spectrometry
tRNA: Transfer RNA
H_2_O_2_: Hydrogen peroxide
NFAT: Nuclear factor of activated T cell
CLL: Chronic lymphocytic leukemia
MT: Mitochondria-targeted
N-AC: N-acetylcysteine
OCR: Oxygen consumption rate
NR: Nicotinamide ribose
NADH: Nicotinamide adenine dinucleotide
scRNA-seq: Single-cell RNA sequencing
CE-ESI–MS: Capillary electrophoresis–electrospray ionization–mass spectrometry
nano-LC–MS: Nanoscale liquid chromatography coupled to mass spectrometry
PEA: Proximity extension assay
CyTOF: Cytometry by time of flight
scATAC-seq: Single-cell assay for transposase-accessible chromatin sequencing
scTCR/BCR-seq: Single-cell paired T/B cell receptor sequencing
PBMC: Peripheral blood mononuclear cell

## References

[1] Zhao H, Wei J, Wei G, Luo Y, Shi J, Cui Q (2020). Pre-transplant MRD negativity predicts favorable outcomes of CAR-T therapy followed by haploidentical HSCT for relapsed/refractory acute lymphoblastic leukemia: a multi-center retrospective study. J Hematol Oncol.

[2] Long AH, Haso WM, Shern JF, Wanhainen KM, Murgai M, Ingaramo M (2015). 4–1BB costimulation ameliorates T cell exhaustion induced by tonic signaling of chimeric antigen receptors. Nat Med.

[3] Wherry EJ, Kurachi M (2015). Molecular and cellular insights into T cell exhaustion. Nat Rev Immunol.

[4] Kasakovski D, Xu L, Li Y (2018). T cell senescence and CAR-T cell exhaustion in hematological malignancies. J Hematol Oncol.

[5] Lemoine J, Ruella M, Houot R (2021). Born to survive: how cancer cells resist CAR T cell therapy. J Hematol Oncol.

[6] Alizadeh D, Wong RA, Yang X, Wang D, Pecoraro JR, Kuo CF (2019). IL15 enhances CAR-T cell antitumor activity by reducing mTORC1 activity and preserving their stem cell memory phenotype. Cancer Immunol Res.

[7] MacIver NJ, Michalek RD, Rathmell JC (2013). Metabolic regulation of T lymphocytes. Annu Rev Immunol.

[8] Vardhana SA, Hwee MA, Berisa M, Wells DK, Yost KE, King B (2020). Impaired mitochondrial oxidative phosphorylation limits the self-renewal of T cells exposed to persistent antigen. Nat Immunol.

[9] Kawalekar OU, O'Connor RS, Fraietta JA, Guo L, McGettigan SE, Posey AD (2016). Distinct signaling of coreceptors regulates specific metabolism pathways and impacts memory development in CAR T cells. Immunity.

[10] Li G, Boucher JC, Kotani H, Park K, Zhang Y, Shrestha B (2018). 4–1BB enhancement of CAR T function requires NF-kappaB and TRAFs. JCI Insight.

[11] Philipson BI, O'Connor RS, May MJ, June CH, Albelda SM, Milone MC (2020). 4–1BB costimulation promotes CAR T cell survival through noncanonical NF-kappaB signaling. Sci Signal.

[12] Pearce EL, Walsh MC, Cejas PJ, Harms GM, Shen H, Wang LS (2009). Enhancing CD8 T-cell memory by modulating fatty acid metabolism. Nature.

[13] Chang CH, Curtis JD, Maggi LB, Faubert B, Villarino AV, O'Sullivan D (2013). Posttranscriptional control of T cell effector function by aerobic glycolysis. Cell.

[14] Chapman NM, Boothby MR, Chi H (2020). Metabolic coordination of T cell quiescence and activation. Nat Rev Immunol.

[15] Wherry EJ (2011). T cell exhaustion. Nat Immunol.

[16] Jadhav RR, Im SJ, Hu B, Hashimoto M, Li P, Lin JX (2019). Epigenetic signature of PD-1+ TCF1+ CD8 T cells that act as resource cells during chronic viral infection and respond to PD-1 blockade. Proc Natl Acad Sci U S A.

[17] Beltra JC, Manne S, Abdel-Hakeem MS, Kurachi M, Giles JR, Chen Z (2020). Developmental relationships of four exhausted CD8(+) T cell subsets reveals underlying transcriptional and epigenetic landscape control mechanisms. Immunity..

[18] Hashimoto M, Kamphorst AO, Im SJ, Kissick HT, Pillai RN, Ramalingam SS (2018). CD8 T cell exhaustion in chronic infection and cancer: opportunities for interventions. Annu Rev Med.

[19] Calderon H, Mamonkin M, Guedan S (2020). Analysis of CAR-mediated tonic signaling. Methods Mol Biol.

[20] McLane LM, Abdel-Hakeem MS, Wherry EJ (2019). CD8 T cell exhaustion during chronic viral infection and cancer. Annu Rev Immunol.

[21] Liu X, Wang Y, Lu H, Li J, Yan X, Xiao M (2019). Genome-wide analysis identifies NR4A1 as a key mediator of T cell dysfunction. Nature.

[22] Wang X, He Q, Shen H, Xia A, Tian W, Yu W (2019). TOX promotes the exhaustion of antitumor CD8(+) T cells by preventing PD1 degradation in hepatocellular carcinoma. J Hepatol.

[23] Alfei F, Kanev K, Hofmann M, Wu M, Ghoneim HE, Roelli P (2019). TOX reinforces the phenotype and longevity of exhausted T cells in chronic viral infection. Nature.

[24] Chen Z, Ji Z, Ngiow SF, Manne S, Cai Z, Huang AC (2019). TCF-1-centered transcriptional network drives an effector versus exhausted CD8 T cell-fate decision. Immunity.

[25] Martinez GJ, Pereira RM, Aijo T, Kim EY, Marangoni F, Pipkin ME (2015). The transcription factor NFAT promotes exhaustion of activated CD8(+) T cells. Immunity.

[26] Lee DW, Kochenderfer JN, Stetler-Stevenson M, Cui YK, Delbrook C, Feldman SA (2015). T cells expressing CD19 chimeric antigen receptors for acute lymphoblastic leukaemia in children and young adults: a phase 1 dose-escalation trial. Lancet.

[27] Maude SL, Laetsch TW, Buechner J, Rives S, Boyer M, Bittencourt H (2018). Tisagenlecleucel in children and young adults with B-cell lymphoblastic leukemia. N Engl J Med.

[28] Hu Y, Wu Z, Luo Y, Shi J, Yu J, Pu C (2017). Potent anti-leukemia activities of chimeric antigen receptor-modified T cells against CD19 in Chinese patients with relapsed/refractory acute lymphocytic leukemia. Clin Cancer Res.

[29] Finney OC, Brakke HM, Rawlings-Rhea S, Hicks R, Doolittle D, Lopez M (2019). CD19 CAR T cell product and disease attributes predict leukemia remission durability. J Clin Invest.

[30] Fraietta JA, Lacey SF, Orlando EJ, Pruteanu-Malinici I, Gohil M, Lundh S (2018). Determinants of response and resistance to CD19 chimeric antigen receptor (CAR) T cell therapy of chronic lymphocytic leukemia. Nat Med.

[31] Xu Y, Zhang M, Ramos CA, Durett A, Liu E, Dakhova O (2014). Closely related T-memory stem cells correlate with in vivo expansion of CAR.CD19-T cells and are preserved by IL-7 and IL-15. Blood.

[32] Yu YR, Imrichova H, Wang H, Chao T, Xiao Z, Gao M (2020). Disturbed mitochondrial dynamics in CD8(+) TILs reinforce T cell exhaustion. Nat Immunol.

[33] Ogando J, Saez ME, Santos J, Nuevo-Tapioles C, Gut M, Esteve-Codina A (2019). PD-1 signaling affects cristae morphology and leads to mitochondrial dysfunction in human CD8(+) T lymphocytes. J Immunother Cancer.

[34] Quintana A, Schwindling C, Wenning AS, Becherer U, Rettig J, Schwarz EC (2007). T cell activation requires mitochondrial translocation to the immunological synapse. Proc Natl Acad Sci U S A.

[35] Junker C, Hoth M (2011). Immune synapses: mitochondrial morphology matters. EMBO J.

[36] Quintana A, Kummerow C, Junker C, Becherer U, Hoth M (2009). Morphological changes of T cells following formation of the immunological synapse modulate intracellular calcium signals. Cell Calcium.

[37] Schwindling C, Quintana A, Krause E, Hoth M (2010). Mitochondria positioning controls local calcium influx in T cells. J Immunol.

[38] Friedman JR, Lackner LL, West M, DiBenedetto JR, Nunnari J, Voeltz GK (2011). ER tubules mark sites of mitochondrial division. Science.

[39] Bantug GR, Fischer M, Grahlert J, Balmer ML, Unterstab G, Develioglu L (2018). Mitochondria-endoplasmic reticulum contact sites function as immunometabolic hubs that orchestrate the rapid recall response of memory CD8(+) T cells. Immunity.

[40] Yog R, Barhoumi R, McMurray DN, Chapkin RS (2010). n-3 polyunsaturated fatty acids suppress mitochondrial translocation to the immunologic synapse and modulate calcium signaling in T cells. J Immunol.

[41] Chan DC (2012). Fusion and fission: interlinked processes critical for mitochondrial health. Annu Rev Genet.

[42] Lackner LL (2014). Shaping the dynamic mitochondrial network. BMC Biol.

[43] Mishra P, Carelli V, Manfredi G, Chan DC (2014). Proteolytic cleavage of Opa1 stimulates mitochondrial inner membrane fusion and couples fusion to oxidative phosphorylation. Cell Metab.

[44] Buck MD, O'Sullivan D, Klein Geltink RI, Curtis JD, Chang CH, Sanin DE (2016). Mitochondrial dynamics controls T cell fate through metabolic programming. Cell.

[45] Cassidy-Stone A, Chipuk JE, Ingerman E, Song C, Yoo C, Kuwana T (2008). Chemical inhibition of the mitochondrial division dynamin reveals its role in Bax/Bak-dependent mitochondrial outer membrane permeabilization. Dev Cell.

[46] Baixauli F, Martin-Cofreces NB, Morlino G, Carrasco YR, Calabia-Linares C, Veiga E (2011). The mitochondrial fission factor dynamin-related protein 1 modulates T-cell receptor signalling at the immune synapse. EMBO J.

[47] Zhang H, Wang P, Bisetto S, Yoon Y, Chen Q, Sheu SS (2017). A novel fission-independent role of dynamin-related protein 1 in cardiac mitochondrial respiration. Cardiovasc Res.

[48] Toyama EQ, Herzig S, Courchet J, Lewis TL, Loson OC, Hellberg K (2016). Metabolism. AMP-activated protein kinase mediates mitochondrial fission in response to energy stress. Science.

[49] Simula L, Pacella I, Colamatteo A, Procaccini C, Cancila V, Bordi M (2018). Drp1 controls effective T cell immune-surveillance by regulating T cell migration, proliferation, and cMyc-dependent metabolic reprogramming. Cell Rep.

[50] Liu YN, Yang JF, Huang DJ, Ni HH, Zhang CX, Zhang L (2020). Hypoxia induces mitochondrial defect that promotes T cell exhaustion in tumor microenvironment through MYC-regulated pathways. Front Immunol.

[51] Varanita T, Soriano ME, Romanello V, Zaglia T, Quintana-Cabrera R, Semenzato M (2015). The OPA1-dependent mitochondrial cristae remodeling pathway controls atrophic, apoptotic, and ischemic tissue damage. Cell Metab.

[52] Sukumar M, Liu J, Mehta GU, Patel SJ, Roychoudhuri R, Crompton JG (2016). Mitochondrial membrane potential identifies cells with enhanced stemness for cellular therapy. Cell Metab.

[53] Siska PJ, Beckermann KE, Mason FM, Andrejeva G, Greenplate AR, Sendor AB (2017). Mitochondrial dysregulation and glycolytic insufficiency functionally impair CD8 T cells infiltrating human renal cell carcinoma. JCI Insight.

[54] Chao T, Wang H, Ho PC (2017). Mitochondrial control and guidance of cellular activities of T cells. Front Immunol.

[55] Summer R, Shaghaghi H, Schriner D, Roque W, Sales D, Cuevas-Mora K (2019). Activation of the mTORC1/PGC-1 axis promotes mitochondrial biogenesis and induces cellular senescence in the lung epithelium. Am J Physiol Lung Cell Mol Physiol.

[56] Tian L, Cao W, Yue R, Yuan Y, Guo X, Qin D (2019). Pretreatment with Tilianin improves mitochondrial energy metabolism and oxidative stress in rats with myocardial ischemia/reperfusion injury via AMPK/SIRT1/PGC-1 alpha signaling pathway. J Pharmacol Sci.

[57] Akimoto T, Pohnert SC, Li P, Zhang M, Gumbs C, Rosenberg PB (2005). Exercise stimulates Pgc-1alpha transcription in skeletal muscle through activation of the p38 MAPK pathway. J Biol Chem.

[58] Jornayvaz FR, Shulman GI (2010). Regulation of mitochondrial biogenesis. Essays Biochem.

[59] Bengsch B, Johnson AL, Kurachi M, Odorizzi PM, Pauken KE, Attanasio J (2016). Bioenergetic insufficiencies due to metabolic alterations regulated by the inhibitory receptor PD-1 are an early driver of CD8(+) T cell exhaustion. Immunity.

[60] Scharping NE, Menk AV, Moreci RS, Whetstone RD, Dadey RE, Watkins SC (2016). The tumor microenvironment represses T cell mitochondrial biogenesis to drive intratumoral T cell metabolic insufficiency and dysfunction. Immunity.

[61] Dumauthioz N, Tschumi B, Wenes M, Marti B, Wang H, Franco F (2021). Enforced PGC-1alpha expression promotes CD8 T cell fitness, memory formation and antitumor immunity. Cell Mol Immunol.

[62] Schlie K, Westerback A, DeVorkin L, Hughson LR, Brandon JM, MacPherson S (2015). Survival of effector CD8+ T cells during influenza infection is dependent on autophagy. J Immunol.

[63] DeVorkin L, Pavey N, Carleton G, Comber A, Ho C, Lim J (2019). Autophagy regulation of metabolism is required for CD8(+) T cell anti-tumor immunity. Cell Rep.

[64] Arbogast F, Arnold J, Hammann P, Kuhn L, Chicher J, Murera D (2019). ATG5 is required for B cell polarization and presentation of particulate antigens. Autophagy.

[65] Xu X, Araki K, Li S, Han JH, Ye L, Tan WG (2014). Autophagy is essential for effector CD8(+) T cell survival and memory formation. Nat Immunol.

[66] Li C, Capan E, Zhao Y, Zhao J, Stolz D, Watkins SC (2006). Autophagy is induced in CD4+ T cells and important for the growth factor-withdrawal cell death. J Immunol.

[67] Kovacs JR, Li C, Yang Q, Li G, Garcia IG, Ju S (2012). Autophagy promotes T-cell survival through degradation of proteins of the cell death machinery. Cell Death Differ.

[68] Gupta SS, Sharp R, Hofferek C, Kuai L, Dorn GW, Wang J (2019). NIX-mediated mitophagy promotes effector memory formation in antigen-specific CD8(+) T cells. Cell Rep.

[69] Glick D, Barth S, Macleod KF (2010). Autophagy: cellular and molecular mechanisms. J Pathol.

[70] Nakatogawa H, Suzuki K, Kamada Y, Ohsumi Y (2009). Dynamics and diversity in autophagy mechanisms: lessons from yeast. Nat Rev Mol Cell Biol.

[71] van der Windt GJ, Everts B, Chang CH, Curtis JD, Freitas TC, Amiel E (2012). Mitochondrial respiratory capacity is a critical regulator of CD8+ T cell memory development. Immunity.

[72] Geltink RIK, Kyle RL, Pearce EL (2018). Unraveling the complex interplay between T cell metabolism and function. Annu Rev Immunol.

[73] Sukumar M, Liu J, Ji Y, Subramanian M, Crompton JG, Yu Z (2013). Inhibiting glycolytic metabolism enhances CD8+ T cell memory and antitumor function. J Clin Invest.

[74] Pearce EL, Pearce EJ (2013). Metabolic pathways in immune cell activation and quiescence. Immunity.

[75] Li W, Qiu S, Chen J, Jiang S, Chen W, Jiang J (2020). Chimeric antigen receptor designed to prevent ubiquitination and downregulation showed durable antitumor efficacy. Immunity.

[76] Patsoukis N, Bardhan K, Chatterjee P, Sari D, Liu B, Bell LN (2015). PD-1 alters T-cell metabolic reprogramming by inhibiting glycolysis and promoting lipolysis and fatty acid oxidation. Nat Commun.

[77] Fisicaro P, Barili V, Montanini B, Acerbi G, Ferracin M, Guerrieri F (2017). Targeting mitochondrial dysfunction can restore antiviral activity of exhausted HBV-specific CD8 T cells in chronic hepatitis B. Nat Med.

[78] Hindupur SK, Gonzalez A, Hall MN (2015). The opposing actions of target of rapamycin and AMP-activated protein kinase in cell growth control. Cold Spring Harb Perspect Biol..

[79] Werlen G, Jain R, Jacinto E (2021). MTOR signaling and metabolism in early T cell development. Genes (Basel).

[80] Hukelmann JL, Anderson KE, Sinclair LV, Grzes KM, Murillo AB, Hawkins PT (2016). The cytotoxic T cell proteome and its shaping by the kinase mTOR. Nat Immunol.

[81] Pollizzi KN, Patel CH, Sun IH, Oh MH, Waickman AT, Wen J (2015). mTORC1 and mTORC2 selectively regulate CD8(+) T cell differentiation. J Clin Invest.

[82] Herzig S, Shaw RJ (2018). AMPK: guardian of metabolism and mitochondrial homeostasis. Nat Rev Mol Cell Biol.

[83] Wang R, Dillon CP, Shi LZ, Milasta S, Carter R, Finkelstein D (2011). The transcription factor Myc controls metabolic reprogramming upon T lymphocyte activation. Immunity.

[84] Gnanaprakasam JNR, Sherman JW, Wang R (2017). MYC and HIF in shaping immune response and immune metabolism. Cytokine Growth Factor Rev.

[85] Zhang L, Romero P (2018). Metabolic control of CD8(+) T cell fate decisions and antitumor immunity. Trends Mol Med.

[86] Yang L, Venneti S, Nagrath D (2017). Glutaminolysis: a hallmark of cancer metabolism. Annu Rev Biomed Eng.

[87] Mullen AR, Hu Z, Shi X, Jiang L, Boroughs LK, Kovacs Z (2014). Oxidation of alpha-ketoglutarate is required for reductive carboxylation in cancer cells with mitochondrial defects. Cell Rep.

[88] Carr EL, Kelman A, Wu GS, Gopaul R, Senkevitch E, Aghvanyan A (2010). Glutamine uptake and metabolism are coordinately regulated by ERK/MAPK during T lymphocyte activation. J Immunol.

[89] Nabe S, Yamada T, Suzuki J, Toriyama K, Yasuoka T, Kuwahara M (2018). Reinforce the antitumor activity of CD8(+) T cells via glutamine restriction. Cancer Sci.

[90] Johnson MO, Wolf MM, Madden MZ, Andrejeva G, Sugiura A, Contreras DC (2018). Distinct regulation of Th17 and Th1 cell differentiation by glutaminase-dependent metabolism. Cell.

[91] Ron-Harel N, Santos D, Ghergurovich JM, Sage PT, Reddy A, Lovitch SB (2016). Mitochondrial biogenesis and proteome remodeling promote one-carbon metabolism for T cell activation. Cell Metab.

[92] Tibbetts AS, Appling DR (2010). Compartmentalization of Mammalian folate-mediated one-carbon metabolism. Annu Rev Nutr.

[93] Newman AC, Maddocks ODK (2017). One-carbon metabolism in cancer. Br J Cancer.

[94] Ma EH, Bantug G, Griss T, Condotta S, Johnson RM, Samborska B (2017). Serine is an essential metabolite for effector T cell expansion. Cell Metab.

[95] Zorov DB, Juhaszova M, Sollott SJ (2014). Mitochondrial reactive oxygen species (ROS) and ROS-induced ROS release. Physiol Rev.

[96] Holmström KM, Finkel T (2014). Cellular mechanisms and physiological consequences of redox-dependent signalling. Nat Rev Mol Cell Biol.

[97] Sena LA, Li S, Jairaman A, Prakriya M, Ezponda T, Hildeman DA (2013). Mitochondria are required for antigen-specific T cell activation through reactive oxygen species signaling. Immunity.

[98] Laniewski NG, Grayson JM (2004). Antioxidant treatment reduces expansion and contraction of antigen-specific CD8+ T cells during primary but not secondary viral infection. J Virol.

[99] Hildeman DA, Mitchell T, Kappler J, Marrack P (2003). T cell apoptosis and reactive oxygen species. J Clin Invest.

[100] Khan O, Giles JR, McDonald S, Manne S, Ngiow SF, Patel KP (2019). TOX transcriptionally and epigenetically programs CD8(+) T cell exhaustion. Nature.

[101] Baldelli S, Aquilano K, Ciriolo MR (2014). PGC-1α buffers ROS-mediated removal of mitochondria during myogenesis. Cell Death Dis.

[102] Scharping NE, Rivadeneira DB, Menk AV, Vignali PDA, Ford BR, Rittenhouse NL (2021). Mitochondrial stress induced by continuous stimulation under hypoxia rapidly drives T cell exhaustion. Nat Immunol.

[103] Zhu L, Kong Y, Zhang J, Claxton DF, Ehmann WC, Rybka WB (2017). Blimp-1 impairs T cell function via upregulation of TIGIT and PD-1 in patients with acute myeloid leukemia. J Hematol Oncol.

[104] Villena JA (2015). New insights into PGC-1 coactivators: redefining their role in the regulation of mitochondrial function and beyond. Febs j.

[105] Frauwirth KA, Riley JL, Harris MH, Parry RV, Rathmell JC, Plas DR (2002). The CD28 signaling pathway regulates glucose metabolism. Immunity.

[106] Menk AV, Scharping NE, Rivadeneira DB, Calderon MJ, Watson MJ, Dunstane D (2018). 4–1BB costimulation induces T cell mitochondrial function and biogenesis enabling cancer immunotherapeutic responses. J Exp Med.

[107] Brentjens RJ, Riviere I, Park JH, Davila ML, Wang X, Stefanski J (2011). Safety and persistence of adoptively transferred autologous CD19-targeted T cells in patients with relapsed or chemotherapy refractory B-cell leukemias. Blood.

[108] Porter DL, Hwang WT, Frey NV, Lacey SF, Shaw PA, Loren AW (2015). Chimeric antigen receptor T cells persist and induce sustained remissions in relapsed refractory chronic lymphocytic leukemia. Sci Transl Med.

[109] Liao W, Lin JX, Leonard WJ (2013). Interleukin-2 at the crossroads of effector responses, tolerance, and immunotherapy. Immunity.

[110] Kalia V, Sarkar S (2018). Regulation of effector and memory CD8 T cell differentiation by IL-2-A balancing act. Front Immunol.

[111] Liu Y, Zhou N, Zhou L, Wang J, Zhou Y, Zhang T (2021). IL-2 regulates tumor-reactive CD8(+) T cell exhaustion by activating the aryl hydrocarbon receptor. Nat Immunol.

[112] Schluns KS, Lefrancois L (2003). Cytokine control of memory T-cell development and survival. Nat Rev Immunol.

[113] Cieri N, Camisa B, Cocchiarella F, Forcato M, Oliveira G, Provasi E (2013). IL-7 and IL-15 instruct the generation of human memory stem T cells from naive precursors. Blood.

[114] Cui G, Staron MM, Gray SM, Ho PC, Amezquita RA, Wu J (2015). IL-7-induced glycerol transport and TAG synthesis promotes memory CD8+ T cell longevity. Cell.

[115] Loschinski R, Bottcher M, Stoll A, Bruns H, Mackensen A, Mougiakakos D (2018). IL-21 modulates memory and exhaustion phenotype of T-cells in a fatty acid oxidation-dependent manner. Oncotarget.

[116] Batra SA, Rathi P, Guo L, Courtney AN, Fleurence J, Balzeau J (2020). Glypican-3-specific CAR T cells coexpressing IL15 and IL21 have superior expansion and antitumor activity against hepatocellular carcinoma. Cancer Immunol Res.

[117] Araki K, Turner AP, Shaffer VO, Gangappa S, Keller SA, Bachmann MF (2009). mTOR regulates memory CD8 T-cell differentiation. Nature.

[118] Kim EH, Sullivan JA, Plisch EH, Tejera MM, Jatzek A, Choi KY (2012). Signal integration by Akt regulates CD8 T cell effector and memory differentiation. J Immunol.

[119] Zheng W, O'Hear CE, Alli R, Basham JH, Abdelsamed HA, Palmer LE (2018). PI3K orchestration of the in vivo persistence of chimeric antigen receptor-modified T cells. Leukemia.

[120] Petersen CT, Hassan M, Morris AB, Jeffery J, Lee K, Jagirdar N (2018). Improving T-cell expansion and function for adoptive T-cell therapy using ex vivo treatment with PI3Kdelta inhibitors and VIP antagonists. Blood Adv.

[121] Dwyer CJ, Arhontoulis DC, Rangel Rivera GO, Knochelmann HM, Smith AS, Wyatt MM (2020). Ex vivo blockade of PI3K gamma or delta signaling enhances the antitumor potency of adoptively transferred CD8(+) T cells. Eur J Immunol.

[122] Crompton JG, Sukumar M, Roychoudhuri R, Clever D, Gros A, Eil RL (2015). Akt inhibition enhances expansion of potent tumor-specific lymphocytes with memory cell characteristics. Cancer Res.

[123] Zhang L, Tschumi BO, Lopez-Mejia IC, Oberle SG, Meyer M, Samson G (2016). Mammalian target of rapamycin complex 2 controls CD8 T cell memory differentiation in a Foxo1-dependent manner. Cell Rep.

[124] Urak R, Walter M, Lim L, Wong CW, Budde LE, Thomas S (2017). Ex vivo Akt inhibition promotes the generation of potent CD19CAR T cells for adoptive immunotherapy. J Immunother Cancer.

[125] Mousset CM, Hobo W, Ji Y, Fredrix H, De Giorgi V, Allison RD (2018). Ex vivo AKT-inhibition facilitates generation of polyfunctional stem cell memory-like CD8(+) T cells for adoptive immunotherapy. Oncoimmunology.

[126] Zhang Q, Ding J, Sun S, Liu H, Lu M, Wei X (2019). Akt inhibition at the initial stage of CAR-T preparation enhances the CAR-positive expression rate, memory phenotype and in vivo efficacy. Am J Cancer Res.

[127] Klebanoff CA, Crompton JG, Leonardi AJ, Yamamoto TN, Chandran SS, Eil RL (2017). Inhibition of AKT signaling uncouples T cell differentiation from expansion for receptor-engineered adoptive immunotherapy. JCI Insight.

[128] Parry RV, Chemnitz JM, Frauwirth KA, Lanfranco AR, Braunstein I, Kobayashi SV (2005). CTLA-4 and PD-1 receptors inhibit T-cell activation by distinct mechanisms. Mol Cell Biol.

[129] Verma V, Jafarzadeh N, Boi S, Kundu S, Jiang Z, Fan Y (2021). MEK inhibition reprograms CD8(+) T lymphocytes into memory stem cells with potent antitumor effects. Nat Immunol.

[130] Kondo T, Ando M, Nagai N, Tomisato W, Srirat T, Liu B (2020). The NOTCH-FOXM1 axis plays a key role in mitochondrial biogenesis in the induction of human stem cell memory-like CAR-T cells. Cancer Res.

[131] Zhang H, Hu Y, Shao M, Teng X, Jiang P, Wang X (2021). Dasatinib enhances anti-leukemia efficacy of chimeric antigen receptor T cells by inhibiting cell differentiation and exhaustion. J Hematol Oncol.

[132] Weber EW, Parker KR, Sotillo E, Lynn RC, Anbunathan H, Lattin J (2021). Transient rest restores functionality in exhausted CAR-T cells through epigenetic remodeling. Science.

[133] Chamoto K, Chowdhury PS, Kumar A, Sonomura K, Matsuda F, Fagarasan S (2017). Mitochondrial activation chemicals synergize with surface receptor PD-1 blockade for T cell-dependent antitumor activity. Proc Natl Acad Sci U S A.

[134] Sedlackova L, Korolchuk VI (2019). Mitochondrial quality control as a key determinant of cell survival. Biochim Biophys Acta Mol Cell Res.

[135] Bailis W, Shyer JA, Chiorazzi M, Flavell RA (2017). No oxygen? No glucose? No problem: fatty acid catabolism enhances effector CD8(+) TILs. Cancer Cell.

[136] Zhang Y, Kurupati R, Liu L, Zhou XY, Zhang G, Hudaihed A (2017). Enhancing CD8(+) T cell fatty acid catabolism within a metabolically challenging tumor microenvironment increases the efficacy of melanoma immunotherapy. Cancer Cell.

[137] Van Rens GH, Arkell SM, Charlton W, Doesburg W (1988). Primary angle-closure glaucoma among Alaskan Eskimos. Doc Ophthalmol.

[138] Ligtenberg MA, Mougiakakos D, Mukhopadhyay M, Witt K, Lladser A, Chmielewski M (2016). Coexpressed catalase protects chimeric antigen receptor-redirected T cells as well as bystander cells from oxidative stress-induced loss of antitumor activity. J Immunol.

[139] Aoki T, Chong LC, Takata K, Milne K, Hav M, Colombo A (2020). Single-cell transcriptome analysis reveals disease-defining T-cell subsets in the tumor microenvironment of classic hodgkin lymphoma. Cancer Discov.

[140] Wang X, Peticone C, Kotsopoulou E, Gottgens B, Calero-Nieto FJ (2021). Single-cell transcriptome analysis of CAR T-cell products reveals subpopulations, stimulation, and exhaustion signatures. Oncoimmunology.

[141] Boroughs AC, Larson RC, Marjanovic ND, Gosik K, Castano AP, Porter CBM (2020). A distinct transcriptional program in human CAR T cells bearing the 4-1BB signaling domain revealed by scRNA-Seq. Mol Ther.

[142] Sheih A, Voillet V, Hanafi LA, DeBerg HA, Yajima M, Hawkins R (2020). Clonal kinetics and single-cell transcriptional profiling of CAR-T cells in patients undergoing CD19 CAR-T immunotherapy. Nat Commun.

[143] Xiao Z, Dai Z, Locasale JW (2019). Metabolic landscape of the tumor microenvironment at single cell resolution. Nat Commun.

[144] Gubin MM, Esaulova E, Ward JP, Malkova ON, Runci D, Wong P (2018). High-dimensional analysis delineates myeloid and lymphoid compartment remodeling during successful immune-checkpoint cancer therapy. Cell.

[145] Ye L, Park JJ, Dong MB, Yang Q, Chow RD, Peng L (2019). In vivo CRISPR screening in CD8 T cells with AAV-sleeping beauty hybrid vectors identifies membrane targets for improving immunotherapy for glioblastoma. Nat Biotechnol.

[146] Wang D, Prager BC, Gimple RC, Aguilar B, Alizadeh D, Tang H (2021). CRISPR screening of CAR T cells and cancer stem cells reveals critical dependencies for cell-based therapies. Cancer Discov.

[147] Spitzer MH, Nolan GP (2016). Mass cytometry: single cells. Many Features Cell.

[148] Hartmann FJ, Mrdjen D, McCaffrey E, Glass DR, Greenwald NF, Bharadwaj A (2021). Single-cell metabolic profiling of human cytotoxic T cells. Nat Biotechnol.

[149] Hartmann FJ, Bendall SC (2020). Immune monitoring using mass cytometry and related high-dimensional imaging approaches. Nat Rev Rheumatol.

[150] Levine LS, Hiam-Galvez KJ, Marquez DM, Tenvooren I, Madden MZ, Contreras DC (2021). Single-cell analysis by mass cytometry reveals metabolic states of early-activated CD8(+) T cells during the primary immune response. Immunity.

[151] Lombard-Banek C, Moody SA, Nemes P (2016). Single-cell mass spectrometry for discovery proteomics: quantifying translational cell heterogeneity in the 16-cell frog (Xenopus) Embryo. Angew Chem Int Ed Engl.

[152] Lombard-Banek C, Moody SA, Manzini MC, Nemes P (2019). Microsampling capillary electrophoresis mass spectrometry enables single-cell proteomics in complex tissues: developing cell clones in live *Xenopus laevis* and Zebrafish Embryos. Anal Chem.

[153] Zhu Y, Clair G, Chrisler WB, Shen Y, Zhao R, Shukla AK (2018). Proteomic analysis of single mammalian cells enabled by microfluidic nanodroplet sample preparation and ultrasensitive NanoLC-MS. Angew Chem Int Ed Engl.

[154] Dhanasekaran AR, Pearson JL, Ganesan B, Weimer BC (2015). Metabolome searcher: a high throughput tool for metabolite identification and metabolic pathway mapping directly from mass spectrometry and using genome restriction. BMC Bioinform.

[155] Sauer U (2006). Metabolic networks in motion: 13C-based flux analysis. Mol Syst Biol.

[156] Assarsson E, Lundberg M, Holmquist G, Bjorkesten J, Thorsen SB, Ekman D (2014). Homogenous 96-plex PEA immunoassay exhibiting high sensitivity, specificity, and excellent scalability. PLoS ONE.

[157] Macaulay IC, Ponting CP, Voet T (2017). Single-cell multiomics: multiple measurements from single cells. Trends Genet.

[158] Stoeckius M, Hafemeister C, Stephenson W, Houck-Loomis B, Chattopadhyay PK, Swerdlow H (2017). Simultaneous epitope and transcriptome measurement in single cells. Nat Methods.

[159] Mimitou EP, Lareau CA, Chen KY, Zorzetto-Fernandes AL, Hao Y, Takeshima Y (2021). Scalable, multimodal profiling of chromatin accessibility, gene expression and protein levels in single cells. Nat Biotechnol.

[160] Katzenelenbogen Y, Sheban F, Yalin A, Yofe I, Svetlichnyy D, Jaitin DA (2020). Coupled scRNA-Seq and intracellular protein activity reveal an immunosuppressive role of TREM2 in cancer. Cell.

[161] Lu YC, Jia L, Zheng Z, Tran E, Robbins PF, Rosenberg SA (2019). Single-cell transcriptome analysis reveals gene signatures associated with T-cell persistence following adoptive cell therapy. Cancer Immunol Res.

[162] Tu AA, Gierahn TM, Monian B, Morgan DM, Mehta NK, Ruiter B (2019). TCR sequencing paired with massively parallel 3' RNA-seq reveals clonotypic T cell signatures. Nat Immunol.

[163] Zheng Y, Liu X, Le W, Xie L, Li H, Wen W (2020). A human circulating immune cell landscape in aging and COVID-19. Protein Cell.

[164] Ludwig LS, Lareau CA, Ulirsch JC, Christian E, Muus C, Li LH (2019). Lineage tracing in humans enabled by mitochondrial mutations and single-cell genomics. Cell.

[165] Lareau CA, Ludwig LS, Muus C, Gohil SH, Zhao T, Chiang Z (2021). Massively parallel single-cell mitochondrial DNA genotyping and chromatin profiling. Nat Biotechnol.

[166] Liu K, Ji K, Guo L, Wu W, Lu H, Shan P (2014). Mesenchymal stem cells rescue injured endothelial cells in an in vitro ischemia-reperfusion model via tunneling nanotube like structure-mediated mitochondrial transfer. Microvasc Res.

[167] Saha T, Dash C, Jayabalan R, Khiste S, Kulkarni A, Kurmi K (2022). Intercellular nanotubes mediate mitochondrial trafficking between cancer and immune cells. Nat Nanotechnol.

[168] Meng G, Li B, Chen A, Zheng M, Xu T, Zhang H (2020). Targeting aerobic glycolysis by dichloroacetate improves Newcastle disease virus-mediated viro-immunotherapy in hepatocellular carcinoma. Br J Cancer.

[169] Kagoya Y, Nakatsugawa M, Yamashita Y, Ochi T, Guo T, Anczurowski M (2016). BET bromodomain inhibition enhances T cell persistence and function in adoptive immunotherapy models. J Clin Invest.

[170] Chowdhury PS, Chamoto K, Kumar A, Honjo T (2018). PPAR-induced fatty acid oxidation in t cells increases the number of tumor-reactive CD8(+) T cells and facilitates anti-PD-1 therapy. Cancer Immunol Res.

[171] Deng Q, Han G, Puebla-Osorio N, Ma MCJ, Strati P, Chasen B (2020). Characteristics of anti-CD19 CAR T cell infusion products associated with efficacy and toxicity in patients with large B cell lymphomas. Nat Med.

[172] Ahl PJ, Hopkins RA, Xiang WW, Au B, Kaliaperumal N, Fairhurst AM (2020). Met-flow, a strategy for single-cell metabolic analysis highlights dynamic changes in immune subpopulations. Commun Biol.

[173] Wagner A, Wang C, Fessler J, DeTomaso D, Avila-Pacheco J, Kaminski J (2021). Metabolic modeling of single Th17 cells reveals regulators of autoimmunity. Cell.

